# Angiotensin-Converting Enzyme Inhibitor-Induced Angioedema Following Long-Term Lisinopril Use: Response to C1 Esterase Inhibitor Treatment

**DOI:** 10.7759/cureus.91346

**Published:** 2025-08-31

**Authors:** Zaid Mula Hussein, Mohammed Emran Khan, Ashfaque Sorathia, Kithsri Karunatilake

**Affiliations:** 1 Geriatrics, William Harvey Hospital, East Kent Hospitals University NHS Foundation Trust, Ashford, GBR; 2 Geriatric Medicine, William Harvey Hospital, East Kent Hospitals University NHS Foundation Trust, Ashford, GBR

**Keywords:** ace inhibitor-induced angioedema, angioedema management, c1 esterase inhibitor therapy, delayed-onset angioedema, drug-induced angioedema, icatibant treatment, medication adverse effects

## Abstract

Angiotensin-converting enzyme inhibitors (ACEIs) are widely prescribed for cardiovascular and renal conditions, but a rare and potentially life-threatening complication is ACEI-induced angioedema, which results from bradykinin accumulation leading to increased vascular permeability and tissue swelling. This condition may present abruptly, even after years of uneventful therapy, and can threaten the airway. We report the case of an elderly woman in her 80s with hypertension, rheumatoid arthritis, and osteoarthritis, who presented with acute throat and tongue swelling, dysphagia, nausea, and hoarseness after 16 years of lisinopril use. She had no fever, stridor, or rash, but recalled two prior self-limiting episodes of similar swelling. Examination showed pronounced left-sided tongue and submandibular swelling, stable vital signs, and no respiratory distress. Laboratory workup revealed mild anemia and raised CRP, but normal white cell count and organ function. Flexible nasoendoscopy showed arytenoid oedema with a patent airway and normal vocal cord movement. In the absence of urticaria, infection, or allergen exposure, a diagnosis of ACEI-induced angioedema was made. Lisinopril was discontinued, and she received icatibant, intravenous corticosteroids, and antihistamines. Mild oxygen desaturation prompted administration of intravenous C1 esterase inhibitor, which led to rapid improvement. The patient recovered overnight, with resolution confirmed on repeat endoscopy, and was discharged after two days with instructions for alternative antihypertensive management. This case emphasizes that ACEI-induced angioedema can occur unpredictably and may not respond to conventional therapies; however, targeted agents such as icatibant and C1 esterase inhibitors can be effective. Early recognition and multidisciplinary intervention are key to preventing airway compromise in these patients.

## Introduction

An older adult presented with acute-onset throat and tongue swelling after 16 years of lisinopril use. Despite no recent medication changes or allergic triggers, clinical assessment suggested angiotensin-converting enzyme (ACE) inhibitor-induced angioedema. Flexible nasal endoscopy confirmed laryngeal oedema. Initial management with icatibant, corticosteroids, and antihistamines provided partial relief, with full resolution following administration of a C1 esterase inhibitor. This case underscores the importance of recognising atypical, delayed-onset angioedema in patients on long-term ACE inhibitor therapy and supports the evolving role of C1 esterase inhibitors in treatment.

Background

ACE inhibitors are widely prescribed for cardiovascular and renal conditions. Though generally well tolerated, ACE inhibitor-induced angioedema is a rare, unpredictable adverse effect that may present without warning, even after years of uneventful use [1-3]. This form of angioedema results from bradykinin accumulation due to ACE inhibition, leading to increased vascular permeability and tissue oedema [2,4]. Prompt identification is critical due to the risk of upper airway obstruction [5]. This case highlights a delayed and severe presentation in an older patient, with notable response to C1 esterase inhibitor therapy [6].

## Case presentation

A woman in her 80s presented to the emergency department with the sudden onset of throat swelling, dysphagia, nausea, and hoarseness, waking her from sleep. There was no history of fever, shortness of breath, stridor, or rash. Notably, she had experienced two prior self-limiting episodes of similar swelling over the past years.

She lives independently with her spouse and is physically active. Her medical history includes hypertension, rheumatoid arthritis, and osteoarthritis. She was taking lisinopril (16 years duration), indapamide, furosemide, mirabegron, pregabalin, leflunomide, and other chronic medications. She had recently initiated folic acid. There were no known allergies or significant family history of angioedema or atopy.

The patient recounted experiencing two previous episodes of similar symptoms a few months prior to the current presentation. On both occasions, she woke up during the night with sudden tongue swelling, which caused significant concern for both herself and her husband. The second episode was notably more severe. At the time, her husband opted for conservative management without seeking immediate medical attention, and the symptoms resolved completely.

During the most recent episode, the patient reported significant tongue swelling that impaired her ability to speak clearly. Her husband promptly called emergency services (999) to seek urgent assistance. However, after waiting for an hour without a response, he decided to transport her to the hospital himself.

Both the patient and her husband expressed considerable anxiety during the event and emphasized their need for urgent medical evaluation. Upon arrival at the emergency department, a physician reviewed her list of regular medications and immediately identified lisinopril as a likely cause. The patient and her husband were surprised by this suggestion, given that she had been taking the medication for approximately 16 years without notable issues.

On initial examination at admission, she was alert and oriented to time, place, and person, with a Glasgow Coma Score of 15/15. Her vital signs were stable with the following parameters: heart rate 76 bpm, blood pressure 185/80 mmHg, respiratory rate 19/min, oxygen saturation 97% on room air, and temperature 36°C. There was marked swelling of the tongue (predominantly on the left side), which was partially occluding the oropharynx. Submandibular swelling was noted with no tenderness. Chest and abdominal examinations were unremarkable. Table 1 summarizes the patient’s blood investigations with corresponding reference ranges.

**Table 1:** Blood investigations.

Test	Result	Reference Range (UK)
Creatinine	72 µmol/L	60-110 µmol/L
eGFR	67 mL/min/1.73m^2^	≥60 mL/min/1.73m^2^
Sodium (Na)	140 mmol/L	133-146 mmol/L
Potassium (K)	4.2 mmol/L	3.5-5.1 mmol/L
Total bilirubin	8 µmol/L	3-17 µmol/L
Alanine transaminase (ALT)	15 U/L	10-50 U/L
Alkaline phosphatase (ALP)	158 U/L	30-130 U/L
Albumin	38 g/L	35-50 g/L
Urea	7.9 mmol/L	2.5-7.8 mmol/L
C-reactive protein (CRP)	25 mg/L	<5 mg/L
Hemoglobin	102 g/L	Male: 130-180 g/L, Female: 115-160 g/L
White cell count	4.6 × 10^9^/L	4.0-11.0 × 10^9^/L
Platelets	242 × 10^9^/L	150-400 × 10^9^/L
Red blood cells (RBC)	3.56 × 10^12^/L	Male: 4.5-6.5 × 10^12^/L, Female: 4.0-5.5 × 10^12^/L
Hematocrit	0.326 L/L	Male: 0.40-0.52 L/L, Female: 0.37-0.47 L/L
Mean corpuscular volume (MCV)	91.6 fL	80-100 fL
Prothrombin time (PT)	13 seconds	9.5-13.5 seconds
Activated partial thromboplastin time (APTT)	25.9 seconds	23-36 seconds

Flexible nasal endoscopy was performed and revealed arytenoid oedema with a patent airway and symmetrical vocal cord movement. The acute onset of oropharyngeal swelling without pruritus or urticaria raised concern for ACE inhibitor-induced angioedema. Differential considerations included allergic/anaphylactic reactions, infectious epiglottitis, and idiopathic angioedema. The absence of allergen exposure, urticarial rash, or infectious symptoms supported a non-histamine-mediated angioedema, most consistent with ACE inhibitor-induced aetiology [1,4].

As part of the management plan, lisinopril was discontinued immediately, and within one to two hours of admission, the patient received subcutaneous icatibant 30 mg, intravenous hydrocortisone 200 mg, intravenous chlorpheniramine 10 mg, and intravenous dexamethasone 6.6 mg.

Three hours later, she developed mild oxygen desaturation and started requiring 2L/min nasal oxygen, prompting administration of 30 mg C1 esterase inhibitor IV. Subsequent clinical and endoscopic evaluations showed significant improvement in oedema, and the patient's oxygen saturation came back to normal [7,8].

The patient remained stable and improved overnight. Repeat flexible nasal endoscopy revealed resolution of mucosal oedema with minimal residual arytenoid swelling. She was discharged two days later with a plan for general practitioner (GP) follow-up to consider alternative antihypertensive treatment.

## Discussion

Angioedema associated with ACE inhibitors affects 0.1-0.7% of users and may develop at any stage of therapy [1-3]. Bradykinin accumulation due to impaired degradation leads to increased vascular permeability, causing oedema [2,4]. Risk factors include older age, female sex, and African ancestry. This patient, although of White ethnicity, had atypical late-onset symptoms after years of lisinopril use, with isolated tongue swelling and two prior self-limiting episodes [9,10].

This case reaffirms the importance of early recognition of ACE inhibitor-induced angioedema. While corticosteroids and antihistamines are often first-line, they are less effective in bradykinin-mediated cases [6]. Icatibant, a bradykinin B2 receptor antagonist and C1 esterase inhibitor, commonly used for hereditary angioedema, has shown efficacy in ACE inhibitor-induced cases [8,10]. In this instance, C1 esterase inhibitor administration correlated with rapid resolution after partial response to earlier therapies [8].

The repeated prior episodes suggest under-recognised prodromal symptoms. Clinicians should consider this diagnosis in patients with unexplained or recurrent swelling who are on ACE inhibitors [1,3,11-14].

In this patient, several factors strongly supported ACE inhibitor-induced angioedema as the most likely diagnosis, rather than allergic, hereditary, acquired, or idiopathic causes. First, the absence of urticaria, pruritus, rash, or systemic allergic features argued against a histamine-mediated (allergic/anaphylactic) angioedema, which typically presents rapidly after allergen exposure and responds to antihistamines and corticosteroids [15]. Second, hereditary or acquired C1-inhibitor deficiency was unlikely, as there was no family history, no history of recurrent abdominal pain or childhood onset swelling, and the patient had reached her 80s without prior angioedema outside of ACE inhibitor therapy. In addition, acquired angioedema (AAE), which is usually associated with serious and/or chronic illnesses such as lymphoproliferative disease, autoimmune conditions, neoplasia, or chronic infections, was not supported by her clinical background, as she had no evidence of such disorders in history, examination, or investigations [16]. Finally, idiopathic recurrent angioedema is a diagnosis of exclusion, but in this case, the two prior self-limiting episodes occurred during ongoing lisinopril use, which is consistent with published evidence that recurrent attacks may precede a more severe episode if the drug is continued [17]. Taken together, the clinical context, lack of allergic, hereditary, or acquired features, temporal association with ACE inhibitor therapy, and resolution after targeted therapy and drug withdrawal all support a diagnosis of ACE inhibitor-induced angioedema.

**Figure 1:**
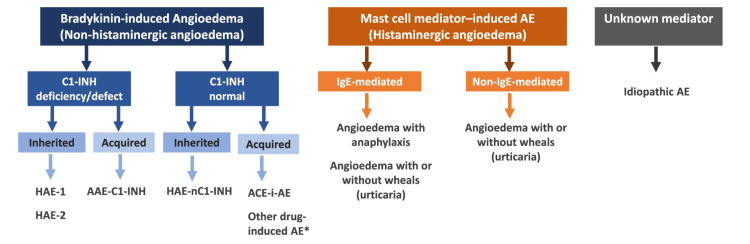
Classification of angioedema. * Other drugs like gliptins, neprilysin inhibitors, or tissue plasminogen activators are thought to potentially induce bradykinin-mediated AE. AE: angioedema; HAE-1: hereditary angioedema due to C1-inhibitor deficiency; HAE-2: hereditary angioedema due to C1-inhibitor dysfunction; AAE-C1-INH: acquired angioedema due to C1-inhibitor deficiency; HAE-nC1-INH: hereditary angioedema with normal C1-Inhibitor levels; ACEI-AE: angiotensin-converting enzyme inhibitor-induced angioedema; IgE: immunoglobulin E Credit: Reference [18]

Angioedema can be classified according to mediator and underlying mechanism as shown in Figure 1 [18,19].

Table 2 summarizes key details from reported cases of ACE inhibitor-induced angioedema.

**Table 2:** Summary of ACEI-induced angioedema case reports. ACEI: angiotensin-converting enzyme inhibitor; C1-INH: C1-inhibitor

Title	Year	Patient Demographics	Clinical Presentation	Management	Outcome
Perindopril-induced angioedema of the lips and tongue [14].	2018	65-year-old Saudi man	Lip and tongue swelling, dysphagia	Antihistamines, corticosteroids, and drug discontinuation	Full resolution, discharged in one day, no recurrence
Angioedema induced by ramipril [20].	2024	70-year-old Indian man	Facial swelling	Antihistamines, steroids, and drug discontinuation	Resolution in two days, no recurrence over a two-year follow-up
ACEI-induced angioedema with airway involvement [21].	2011	76-year-old woman	Tongue swelling, airway oedema, fever, raised inflammatory markers	Steroids, antibiotics, and intubation	Extubated on day 4, discharged from ICU on day 5
ACEI-associated angioedema [22].	2011	78-year-old woman	Macroglossia, airway obstruction, previous episode	Adrenaline, corticosteroids, and intubation	Fatal outcome after complications
Icatibant compared to steroids and antihistamines [23].	2017	Adults 18-95 years, all White individuals	ACEI-induced angioedema	Icatibant vs. steroids + antihistamines	Faster resolution with icatibant (8h vs. 27.1h), fewer rescue treatments
ACEI-induced angioedema: allergy clinic review [24].	2010	Nine patients, mean age 63.4 years, mostly male	Lips, tongue, larynx, and face swelling	Steroids, antihistamines; some ICU admissions	Six life-threatening, one tracheostomy
Effectiveness of C1-INH therapy in ACEI-induced angioedema [25].	2021	Nine patients, mostly female, predominantly Caucasian individuals, one Filipino individual	Upper airway or facial angioedema, including lips, tongue, soft palate, and cheeks	C1-INH concentrate (1000-1500 IU); some received multiple doses; initial treatment with steroids, epinephrine, and antihistamines	Mixed results; five resolved within ~13.5h, others within 22-72h; one recurrence, mostly required intubation

The summary table of ACE inhibitor-induced angioedema case reports highlights the variable clinical presentations, management strategies, and outcomes associated with this potentially life-threatening adverse effect. Most cases required discontinuation of the offending ACE inhibitor along with supportive treatment such as antihistamines, corticosteroids, or intubation in severe cases. While some patients experienced rapid resolution without recurrence, others faced significant complications, including ICU admission, tracheostomy, or even death. Notably, newer therapies like icatibant and C1-inhibitor (C1-INH) showed promise in select cases, though outcomes varied. This underscores the importance of early recognition and individualized management in improving prognosis.

In this case, the decision to escalate to C1 esterase inhibitor was prompted by clinical deterioration (oxygen desaturation) after partial response to icatibant. While there is no universally accepted protocol for sequencing therapies in ACE inhibitor-induced angioedema, the availability of C1-INH at our hospital, clinical decision, and published evidence of benefit in bradykinin-mediated angioedema supported its use as an escalation strategy [25].

Learning points and take-home messages

ACE inhibitor-induced angioedema can present even after prolonged and previously uneventful use of the medication. Clinicians should be aware that its presentation may be atypical, often manifesting as isolated swelling of the tongue or throat without accompanying urticaria. In severe cases that do not respond to conventional therapies, treatment with C1 esterase inhibitor has been shown to be effective. A history of prior self-limiting episodes of swelling in patients on ACE inhibitors should raise suspicion for this diagnosis. Early involvement of a multidisciplinary team is essential to promptly recognize and manage the condition, thereby preventing potentially life-threatening airway compromise.

## Conclusions

This case underscores the critical importance of maintaining a high index of suspicion for ACE inhibitor-induced angioedema, even in patients with many years of uneventful medication use. The absence of urticaria or identifiable allergic triggers can delay diagnosis, placing patients at significant risk of airway compromise. Timely recognition, immediate discontinuation of the offending agent, and appropriate intervention with targeted therapies, such as C1 esterase inhibitor, can dramatically alter outcomes, as demonstrated by the rapid clinical improvement in this patient. Moreover, the case highlights the limitations of conventional therapies like corticosteroids and antihistamines in bradykinin-mediated angioedema, emphasizing the evolving role of specific agents such as icatibant and C1-INH. Clinicians must remain vigilant and consider this potentially life-threatening diagnosis in any patient presenting with unexplained oropharyngeal swelling while on ACE inhibitors, regardless of treatment duration.
